# Impact of the COVID-19 pandemic on handwashing practices among community members in the middle belt of Ghana: evidence from a cross-sectional study

**DOI:** 10.11604/pamj.2024.47.122.37914

**Published:** 2024-03-19

**Authors:** Edward Anane Apraku, Sulemana Watara Abubakari, Richard Joshua Tetteh, Samuel Afari-Asiedu, Ekow Samuel Harrison, Francis Agbokey, Solomon Nyame, Mieks Frenken Twumasi, Wisdom Adeapena, Charles Zandoh, Livesy Naafoe Abokyi, Lawrence Gyabaa Febir, Kwaku Poku Asante

**Affiliations:** 1 Kintampo Health Research Centre, Research and Development Division, Ghana Health Service, Kintampo-Bono East Region, Kintampo, Ghana,; 2 Department of Health Services, Policy Planning, Management and Economics, University for Development Studies, Northern Region, Tamale, Ghana

**Keywords:** Handwashing, COVID-19 pandemic, residents, middle belt of Ghana

## Abstract

Handwashing is an effective public health intervention for preventing the spread of coronavirus (COVID-19). Maintenance of clean hands is particularly important during the pandemic, to break the cycle of human-to-human transmission of the virus. This study explored the potential impact of the COVID-19 pandemic on the handwashing behaviours of residents before and during the pandemic. A mixed-method cross-sectional design using standardised questionnaire was used to examine hand handwashing behaviours among residents before and during the COVID-19 pandemic in the middle belt of Ghana. However, this paper reports on the quantitative data on handwashing behaviour only. A total of 517 participants between 18 to 60 years were randomly selected from the Kintampo Health and Demographic Surveillance System (HDSS) database. Descriptive statistics were performed and McNamar test was used to estimate the difference in the handwashing behaviour of residents. Majority of the respondents were females (54.6%). The majority of them 77.0% (398) usually wash their hands with soap and water. Those who washed hands 4 to 6 times a day before the pandemic increased from 39.9% (159) to 43.7% (174). About 34.8% (180) had received training on hand washing and television 53.3% (96) emerged as the main source of training. Ownership of handwashing facilities increased from 11.4% (59) to 22.8% (118) during the pandemic. The odds of handwashing after handshaking were lower 0.64 (95% C1: 0.44-0.92,) during the pandemic. Television (53.3%) was the main source of training for respondents who had received training on handwashing (34.8%). The odds of owning a handwashing facility during the pandemic were 3 times higher than before (OR = 2.97, 95% CI: 1.94 - 4.65). The odds of handwashing after sneezing were 1.8 (95% CI: 1.19-2.92) times higher during the pandemic. Handwashing behaviours during the pandemic improved among residents than before. However, there is a need to intensify health education and media engagement on proper handwashing practices to protect the population against infectious diseases.

## Introduction

Handwashing is an effective public health intervention for preventing the spread of coronavirus (COVID-19) [1]. Maintaining proper handwashing behaviour, particularly during this era of the COVID-19 pandemic is paramount and requires conscious and consistent practice. Water, sanitation, and hygiene (WASH) practices are frequently the first line of defence in infectious disease control including COVID-19 [2]. Maintenance of clean hands is particularly important during the pandemic, to break the cycle of human-to-human transmission of the virus [3]. The campaign for WASH practices during this period of the pandemic heightened when SARS-CoV-2 transmissions were found to be associated with animal-to-human and human-to-human contacts through fomites, faeco-oral contaminants, contaminated droplets/aerosols from exhaled air, coughs, and sneezes that contaminate the immediate surroundings [4,5]. As a result, handwashing with soap and water or use of alcohol rub or sanitizers were among the recommended preventive measures against COVID-19. Handwashing is among the cheapest and essential interventions against infectious diseases such as diarrhoea, cholera, typhoid, and dysentery [6].

A number of World Health Organization (WHO) directives for the control of COVID-19 were put out to protect people against the pandemic [7]. These included vaccination and adherence to local guidelines on immunization. Also, a physical barrier of at least one meter should be maintained between people, even if they do not seem to be unwell. One is expected to put on a mask that fits correctly when unable to physically separate and when the ventilation is inadequate. Crowds and direct touch be avoided, and it is important to clean hands often with a hand rub that contains alcohol or soap and water [8]. Others were covering mouth and nose with a tissue or a bent elbow when coughing or sneezing to prevent the spread of the virus, and immediately throw away any used tissues. Until one recovered, you were to isolate if you have symptoms or if you test positive for COVID-19 [8]. In Ghana, additional measures were put in place to detect, contain, and stop the spread of the disease in 2020 [9]. These included closing schools, churches, mosques, and other places of worship on March 16, 2020; prohibiting entry for visitors from nations where there had been more than 200 confirmed cases of COVID-19 within the previous 14 days on March 17, 2020; requiring all visitors to remain in quarantine for 48 hours before the nation's borders were closed [9].

Despite widespread recognition of the importance of handwashing in preventing COVID-19 infection, access to handwashing facilities such as Veronica buckets stand and items such as alcohol-based hand sanitizer, soap, and water remain limited in communities and health-care facilities, especially in low- and middle-income countries [2]. Due to the heightened campaign for WASH during this era, some communities provided handwashing facilities at various locations such as schools, churches, mosques, workplaces, and sales outlets within communities.

There has been the proliferation of improvised handwashing stations by individuals, households, and non-governmental organizations (NGOs) since the beginning of the pandemic, using empty rubber bottles/cans, and or rubber containers to produce hands-free devices such as tippy taps in many communities. Even though handwashing facilities may be available in some communities in rural Ghana, access and use of these facilities remain a challenge [10]. The determinants of handwashing and the perception of community members in Ghana towards handwashing practices, especially during COVID-19 warrant the need for inquiry. The objective of the study was to explore the potential impact of COVID-19 disease on handwashing behaviours of residents before and during the pandemic.

## Methods

**Study design and setting:** this study adopted a cross-sectional design using a quantitative approach. A quantitative data collection method using a pre-tested self-developed questionnaire was employed to examine hand hygiene behaviours among residents before and during COVID-19 in the middle belt of Ghana between 15^th^ June 2020 to 7^th^ July 2020. The questionnaire was designed using Survey Solutions Version 19.05 and installed on Android tablets for the collection of data [11]. Data validation tests such as branching logic, ranges, and consistency checks were built into the data collection tools' design to verify that all data fields were filled out before being submitted. For the sake of field verification, the Global Positioning System (GPS) coordinates were automatically activated for accurate recordings.

The study was conducted in six (6) adjoining municipalities/districts in the Bono East Region of Ghana, where the Kintampo Health Research Centre (KHRC) maintains a Health and Demographic Surveillance System (HDSS): Kintampo North Municipal (KNM), Kintampo South District (KSD), Techiman South Municipal (TSM), Techiman North District (TND), Nkoranza South Municipal (NSM), and Nkoranza North District (NND). As of 2020, the HDSS covered an estimated total population of about 430,728 people made up of 47.8% males and 52.2% females. The various districts population composition is as follows: KSD (16.3%), KNM (21.6%), NSM (15.7%), NND (6.1%), TSM (26.1%), and TND (14.2%). In the study area, there are 9 hospitals, 35 health centres, and 214 operational Community-based Health Planning and Services (CHPS). The population is predominantly rural and multi-ethnic, with the bulk of people being peasant farmers [12].

**Sample size calculation and distribution:** the sample size was estimated based on a survey on Ebola virus disease knowledge, attitudes, and prevention measures in the Kintampo areas of Ghana, where 83 percent of respondents were aware of the disease [13]. This paper used data on handwashing which was collected as part of a study that sought to assess knowledge, attitude, and preventive practices of COVID-19 infection in the middle belt of Ghana. The questionnaire comprised 9 modules including a module that elicited questions on handwashing before and during COVID-19 and ownership of handwashing facilities. Hence the sampling size was based on the earlier study on Ebola. Thus, using an assumed 0.83 prevalence of knowledge on COVID-19 and handwashing, with a 95% confidence level, and a 0.0324 error margin, a total sample size of 517 was required for the study. The sample size was calculated using the formula:


$$n=\frac{\alpha^{2}p\left(1-p\right)}{\varepsilon^{2}}$$

Where p is the prevalence of the study, α is the significance level and ε is the error margin of the study [14]. The following is a proportional division of the sampling distribution based on the size of each research district: Kintampo North and South (196 (38%)), Nkoranza North and South (112 (22%)), and Techiman North and South (209 (40%)). To avoid sampling bias, a simple random sampling technique was used to select participants from each site (compounds within the study area have compound numbers, and the table of random number method was used to select compounds).

**Eligibility criteria:** all adult resident household members who were of sound mind between the ages of 18 years and above were eligible to participate in the survey. Non-resident members were excluded from the study. These are migrants who do not meet the three-month criteria to be registered as a resident member into the HDSS.

**Data collection procedure:** a structured questionnaire was administered to participants by trained interviewers who have experience in data collection with a minimum of senior high school education and were trained on the data collection tools and study procedures for two weeks. The interviews were conducted face-to-face, observing all COVID-19 control measures. Compounds and households within the catchment area have unique compound and household numbers, and the study used a table of random numbers method to select eligible compounds and households in the study communities. Pretesting of the questionnaire via piloting was performed. Responses to the pre-test and post-test were analysed for consistency. The study also performed validity checks with the help of data managers. During the study, enumerators visited compounds in the communities and interviewed adult participants of selected households based on the simple random method mentioned earlier.

**Data management:** the questionnaire for data collection was designed using Survey Solutions Version 19.05 and installed on Android tablets. Completed data were synchronized daily to a database hosted by the KHRC Computer Centre. Data obtained were crosschecked for consistency, completeness, and accuracy. The data was managed using structured query language (SQL) on a password-protected computer and managed by a trained data manager.

**Data analysis:** STATA version 14.0 was used for statistical analysis. Descriptive statistics such as frequencies and percentages were performed to describe the data and presented in figures and tables. The wealth quintile was computed using principal component analysis where household assets such as ownership of TVs, cars, fridges, tractors, radios, cows, sheep, goats, and land among others were used in the computation.

The number of times that respondents washed their hands in a typical day before the COVID-19 pandemic was used as a proxy for handwashing behaviour before COVID-19. The number of times that respondents washed their hands “yesterday” referring to the day before the interview was also used as a proxy for current handwashing with soap and water. The association between the socio-demographic characteristics of participants and ownership of the handwashing facility was also tested using the Chi^2^ test of association. The McNemar test was used to estimate the difference in handwashing behaviours before and during the pandemic. This test is an appropriate test for paired samples such as before and after samples. It is therefore imperative to use it since we sought to assess the difference in handwashing behaviours among community members before and during the pandemic [15].

**Ethical consideration:** ethics approval was obtained from the KHRC institutional ethics committee (study ID: KHRCIEC/2020-09). An informed consent was obtained from all study participants prior to the commencement of study procedures. The respondents' anonymity was ensured by collecting the data (quantitative and qualitative components) in an enclosed area with no other individuals present. Also, we ensured personal identifiers were not used in any aspect of the write-up. All participants were informed about the study's purpose, rationale, procedures, potential risks, and benefits, and were given the opportunity to ask questions. Participants were advised that participation in the study was entirely voluntary and that they could opt out at any moment with no repercussions. Study participants were not paid for their participation in the study.

## Results

**Sociodemographic characteristics of participants:** a total of 517 respondents made up of 54.6% (282) females and 45.4% (235) males were involved in the study. Respondents ranged from 18 to 60 years of age with an average age of 39 years. About 38.9% (201) of the respondents had no formal education, 17% (88) had attained primary school, and 23.6% (122) had attained Junior high school or middle school education. Most of the respondents representing 43.3% (224) were farmers while 25% (130) were traders or businessmen/women. The majority of the respondents representing 67.9% (351) were married. Data were obtained from all participants as presented in Table 1.

**Table 1:** socio-demographic characteristics of respondents (N=517)

Demographics	Total n (%)
Gender	
Female	282 (54.6)
Male	235 (45.4)
**Age group**	
Less than 30 years	135 (26.1)
30 - 40 years	147 (28.4)
Above 40 years	235 (45.5)
**Level of education**	
None	201 (38.9)
Primary	88 (17.0)
Middle/JSS	122 (23.6)
SHS/post-middle college	90 (17.4)
University	16 (3.1)
**Occupation**	
No employment	62 (12.0)
Clerical/secretarial	8 (1.5)
Employed tradesman	27 (5.2)
Farmer	224 (43.3)
Professional	20 (3.9)
Student	37 (7.2)
Trader/businessman	130 (25.1)
Other	9 (1.7)
**Marital status**	
Married	351 (67.9)
Single	91 (17.6)
Living together	25 (4.8)
Separated	21 (4.1)
Widowed	29 (5.6)
**Religious status**	
Christian	217 (42.0)
Muslim	278 (53.8)
Traditional	8 (1.5)
None	14 (2.7)
**Wealth quintile**	
Very poor	88 (17.0)
More poor	105 (20.3)
Poor	116 (22.4)
Less poor	99 (19.1)
Least poor	109 (21.1)

JSS: junior secondary school; SHS: senior high school

**Figure 1:**
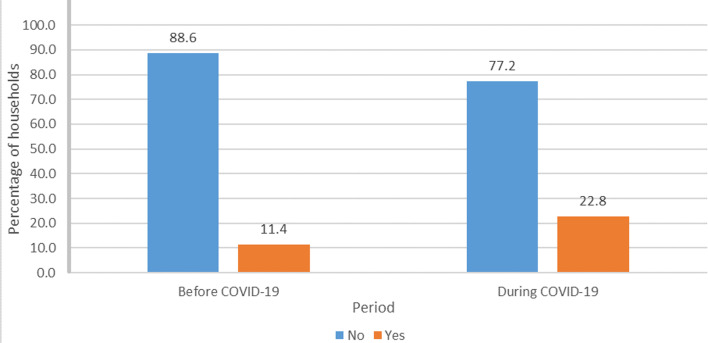
prevalence of household ownership of handwashing facility before and during COVID-19 pandemic

**Household ownership of hand washing facility:** the survey explored whether respondents had handwashing facilities in their households. Out of the total sample, 59 respondents representing 11.4% had handwashing facilities in their households before the COVID-19 pandemic. However, the number of respondents whose households have a designated place for handwashing increased from 11.4% (59) to 22.8% (118) during the COVID-19 pandemic (Figure 1).

**Figure 2:**
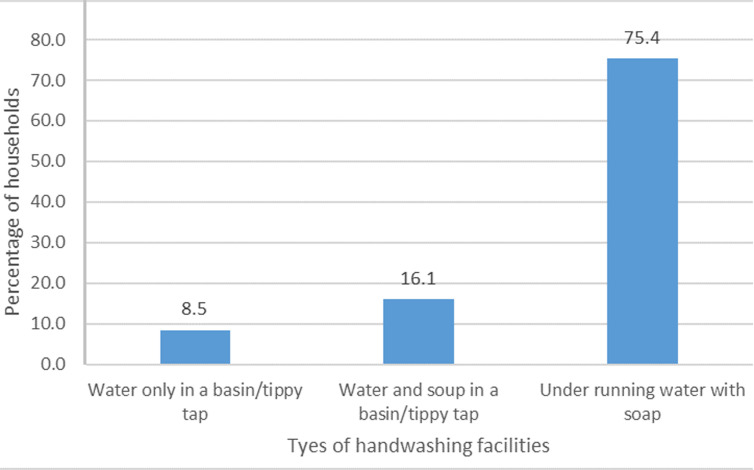
types of handwashing facilities owned by households

**Type of handwashing facility:** data collectors observed the type of handwashing facility owned by the respondent´s households during the time of visit for the interview. Out of the 118 respondents whose household´s had a handwashing facility, the majority 75.4% (89) had running water with a soap facility, and 16.1% (19) of them had a basin of water or tippy tap and soap. The remaining 8.5% (10) had only a basin of water or a tippy tap only (Figure 2).

**Figure 3:**
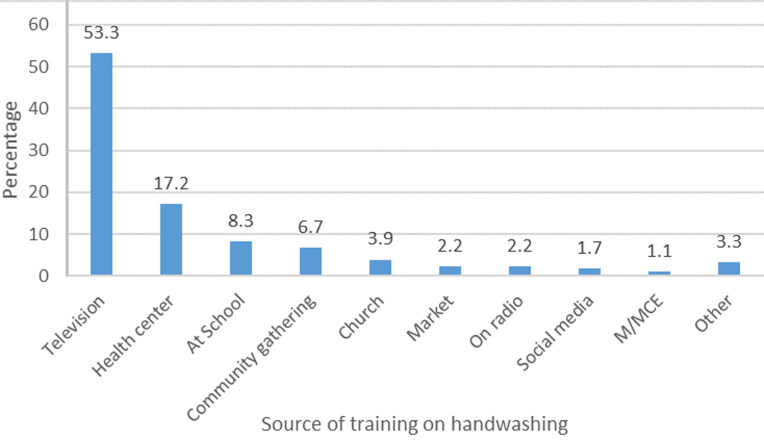
source of training on handwashing during the COVID-19 pandemic

**Training and sources of handwashing training:** respondents were asked whether they had received any form of training on handwashing since the outbreak of the COVID-19 pandemic. Out of 517 respondents interviewed, 34.8% (180) of respondents reported having received training on handwashing, out of the 180 respondents who had any form of training on handwashing, the majority 53.3% (96) identified television as the source of training while 17.2% (31) and 8.3% (15) had received training at health facilities and schools respectively (Figure 3).

**Handwashing with soap (HWWS) before and during COVID-19:** information on respondents´ attitudes towards handwashing specifically with soap was obtained. The majority, 77.0% (398) of the respondents answered that they usually washed their hands with soap. Out of this 77.0%, we further asked of how often they washed their hands with soap on a typical day before the COVID-19 pandemic. About 40% (159) and 37% (150) of respondents indicated that they usually washed their hands with soap between 4 to 6 times and 1 to 3 times respectively in a typical day before the pandemic as shown in Table 1.

The number of times that respondents washed their hands “yesterday” as a proxy for current handwashing with soap was also explored. About 43.7% (174) and 25.9% (103) of the respondents said that they washed their hands with soap between 4 to 6 and 1 to 3 times yesterday respectively as demonstrated in Table 2.

**Table 2:** handwashing with soap before and during COVID-19

Attributes	Total	Frequency	Percent
**Do you usually wash your hands with soap?**			
No	517	119	23
Yes		398	77
**In a typical day before COVID-19, how often did you wash your hands with soap?**			
1 to 3 times	398	150	37.7
4 to 6 times		159	39.9
7 to 10 times		68	17.1
10 or more		21	5.3
**Number of times that you washed your hands with soap yesterday?**			
1 to 3 times	398	103	25.9
4 to 6 times		174	43.7
7 to 10 times		88	22.1
10 or more		33	8.3

The number of respondents who usually washed their hands 4 to 6 times before the pandemic increased from 39.9% to 43.7% during the pandemic suggesting an increase of a 3.8 percentage point difference. Also, respondents who washed their hands with soap between 7 to 10 times before COVID-19 increased from 17.1% to 22.1% during the pandemic as shown in Table 2.

**Impact of handwashing training on handwashing practices:** Table 3 provides information on the impact of training on handwashing on actual handwashing practices. The study examined this association using the Chi-square test. Handwashing practices during the COVID-19 pandemic are measured using nine parameters as shown in Table 3. Findings from the table show that training on handwashing significantly impacts handwashing practices after boarding a bus. However, we see from the table that the remaining eight indicators of handwashing practices are not influenced significantly by training on handwashing. Handwashing after visiting the toilet, sneezing, before and after shaking hands, and meals among the other indicators in Table 3 are not significantly influenced by training on handwashing.

**Table 3:** impact of handwashing training on handwashing practices (N=517)

Attributes	Training on handwashing		p-value
**Usually wash your hands with soap and water?**	**Yes (%)**	**No (%)**	
Yes	146 (81.1)	269 (79.8)	
No	34 (18.8)	68 (20.2)	0.726
**Wash hands after visiting the toilet**			
Yes	165 (91.7)	294 (87.2)	
No	15 (8.3)	43 (12.8)	0.129
**Wash hands after sneezing**			
Yes	42 (23.3)	55 (16.3)	
No	138 (76.7)	282 (83.7)	0.052
**Wash hands before and after meals**			
Yes	170 (94.4)	307 (91.1)	
No	10 (5.6)	30 (8.9)	0.175
**Wash hands before and after handshaking**			
Yes	32 (17.8)	42 (12.5)	
No	148 (82.2)	295 (87.5)	0.1
**Wash hands yesterday after changing a diaper**			
Yes	44 (24.4)	61 (18.1)	
No	136 (75.6)	276 (81.9)	0.088
**Washed hands yesterday after burial**			
Yes	19 (10.6)	32 (9.5)	
No	161 (89.4)	305 (90.1)	0.7
**Washed hands yesterday after boarding the vehicle**			
Yes	36 (20.0)	144 (80.0)	
No	33 (9.8)	304 (86.7)	0.001
**Washed hands yesterday after other activities**			
Yes	35 (19.4)	65 (19.3)	
No	145 (80.7)	272 (80.7)	0.966

**Association between socio-demographic characteristics of participants and ownership of handwashing facility:** Table 4 shows the association between socio-demographic characteristics of handwashing facility. Findings from the table show that educational level significantly influences the ownership of handwashing facilities. However, gender, occupation, religion, and marital status do not influence the ownership of handwashing facilities.

**Table 4:** association between socio-demographic characteristics of participants and ownership of handwashing facility (N=517)

	Ownership of handwashing facility		p-values
**Gender**	**Yes**	**No**	
Male	60 (21.3)	58 (24.7)	
Female	222 (78.7)	177 (75.3)	0.36
**Education level**			
None	168 (42.1)	33 (28.0)	
Primary	67 (16.8)	21 (17.8)	
Middle/JHS	95 (23.8)	27 (22.9)	
SHS/post-secondary	61 (15.3)	29 (24.6)	
Tertiary	8 (2.0)	8 (6.8)	<0.01
**Occupation**			
Not employed	44 (11.0)	18 (15.3)	
Farmer/labourer	185 (46.4)	39 (33.1)	
Professional (teacher, nurse, etc.)	139 (34.8)	46 (39.0)	
Student	25 (6.3)	12 (10.2)	
Other	6 (1.5)	3 (2.5)	0.09
**Religion**			
Christian	157 (39.4)	60 (50.9)	
Muslim	222 (55.6)	56 (47.5)	
Tradition	8 (2.0)	0 (0.0)	
None	12 (3.0)	2 (1.7)	0.07
**Marital status**			
Married	294 (73.7)	82 (69.5)	
Single	66 (16.5)	25 (21.2)	
Separated	39 (9.8)	11 (9.3)	0.51

JHS: junior high school; SHS: senior high school

**Impact of the COVID-19 pandemic on handwashing behaviour:** Table 5 highlights the handwashing behaviour of participants before and during the COVID-19 pandemic in the study population. Handwashing after visiting the toilet reduced by 0.09% (CI: -0.13 - -0.07, p=0.001) during the pandemic. There was a statistically significant difference of 0.09 (p=0.001) in handwashing before and during the pandemic. The odds of handwashing after visiting the toilet were lower by 0.10 (CI: 0.02-0.20, p=0.001) during the pandemic than before. Handwashing after sneezing improved by 0.05% (CI: 0.02-0.09) during the pandemic. The difference in handwashing before and during the pandemic was statistically significant at 0.05 (p=0.003). The odds of handwashing after shaking hands with other people were lower by 0.64 (95% CI: 0.44-0.92) during the pandemic than before. There was a statistically significant difference of 0.37 (p=0.017) in handwashing after handshakes with other people before and during the pandemic.

**Table 5:** impact of the COVID-19 pandemic on handwashing behaviour (McNamer test) (N=517)

Before COVID-19 pandemic		During COVID-19 pandemic			Difference (95% CI)	X^2^	OR (95% CI)	P-value
**Washed hands after visiting the toilet**		**No**	**Yes**	**Total**	**-0.09 (-0.13 - -0.07)**	**44.08**	**0.10 (0.02-0.20)**	0.001***
	No	3	4	7				
	Yes	55	455	510				
	Total	58	459	517				
**Washed hands after sneezing**		**No**	**Yes**	**Total**	**0.05 (0.02-0.09)**	**8.34**	**1.85 (1.19-2.92)**	0.003**
	No	387	61	448				
	Yes	33	36	69				
	Total	420	97	517				
**Washed hands after shaking hands with other people**		**No**	**Yes**	**Total**	**-0.37 (-0.73- -0.23)**	**6.03**	**0.64 (0.44-0.92)**	**0.017**
	No	364	51	415				
	Yes	79	23	102				
	Total	443	74	517				
**Washed hands after changing baby's diaper**		**No**	**Yes**	**Total**	**-0.41 (-0.47- -0.34)**	**108.74**	**0.10 (0.03-0.14)**	0.001***
	No	74	10	84				
	Yes	136	90	226				
	Total	210	100	310				
**Washed hands after burial(cemetery)**		**No**	**Yes**	**Total**	**-0.72 (-0.76- -0.67)**	**281.12**	**0.01(0.01-0.03)**	0.001***
	No	62	3	65				
	Yes	290	44	334				
	Total	352	47	399				
**Ownership of designated handwashing facility**		**No**	**Yes**	**Total**	**0.11 (0.07-0.15)**	**29.25**	**2.97 (1.94-4.65)**	0.001***
	No	369	89	458				
	Yes	30	29	59				
	Total	399	118	517				

** : inference; ***: p<0.01; **: p<0.05; *: p<0.1

The odds of handwashing after sneezing were 1.8 (95%CI: 1.19-2.92) times higher during the pandemic than before.

Handwashing after changing baby diapers was reduced by 41% (95%CI: -0.47- -0.34) during the time of the pandemic. The difference (0.41) in handwashing after changing baby diapers was statistically significant (p=0.001) before and during the pandemic. The odds of handwashing after changing a baby's diaper were lower by 0.10 (95%CI: 0.03-0.14) during the pandemic than before. Handwashing after burial was reduced by 72% (95%CI: -0.76- -0.67) during the pandemic and this change or difference (0.72) was statistically significant (p=0.001). Ownership of handwashing facilities increased by 11% (95%CI: 0.07-0.15) during the pandemic, the increment (95%CI: 0.07-0.15) was statistically significant (p=0.001). The odds of owning a handwashing facility during the pandemic were about 3 times higher than before the pandemic (OR = 2.97, 95% CI: 1.94 - 4.65) as shown in Table 5.

## Discussion

The study set out to assess the impact of the COVID-19 pandemic on handwashing behaviours prior to and during the pandemic. Hand hygiene is considered to be one of the most significant control measures for reducing COVID-19 infection in both community and clinical settings [2]. To reduce the spread of the COVID-19 virus, proper handwashing is critical [16]. Creating awareness and training on handwashing is very essential, especially in deprived communities or settings where handwashing facilities may be limited or inadequate.

Training on proper handwashing as presented by the current study was relatively low (34.8%) among the surveyed respondents. In order to ensure effective handwashing practices, efforts should be geared towards providing key steps to proper handwashing [2]. How effectively one washes his/her hands, especially during this pandemic era may have implications on infection risk. Given the number of people trained in handwashing as reported by this study suggests a need for policymakers to target interventions towards handwashing training amongst residents. Some of these interventions could be channelled through television as it was found to be the highest source of training on handwashing in this study. It could be complemented with training at the health centres or at schools to reach more people. The content of the message should be clear and actionable to improve handwashing and sanitation behaviours.

The impact of the COVID-19 pandemic on handwashing after visiting the toilet saw a reduction instead of an improvement. This may be explained by the fact that during this pandemic era, other people resorted to using hand sanitizers, which may have affected the results. This may also be applicable to the results on the impact of the COVID-19 pandemic on handwashing after changing baby diapers. Such that, those who had children and were using diapers before the pandemic may have transitioned to a stage where their children were not using diapers any longer.

Ownership of handwashing facilities before the pandemic as reported by the current study was very low (11.4%). However, this finding contradicts findings of the Ghana Multiple Indicator Cluster Survey (MICS) study results which elaborates that households in the rural part of Ghana with handwashing facilities where soap and water are observed is 41.8% [17]. This contradiction may be due to the fact that all rural communities in the country were surveyed while the current study observed selected rural areas in the middle belt of Ghana only. In addition, a study by To *et al*. (2016) [13] in Vietnam reported that about 98% of households had a specific place for handwashing. Although the number of households who had handwashing facilities doubled during the pandemic, it is however still on the low side and presents a challenge in the fight to prevent infections. To curb the situation and encourage ownership of handwashing facilities, there is a need for the deployment of low-cost facilities to improve access [2]. The emergence of the COVID-19 pandemic influenced some households to own handwashing facilities and the change was statistically significant. This emphasizes the fact that the pandemic has positively impacted or changed the behaviour of some households toward ownership of handwashing facilities during the pandemic.

Most of the households had running water and soap as part of their handwashing facility, which was encouraging. There is the need to encourage those who used water in a basin or tippy tap only to add soap. Achieving this may enhance the effectiveness of handwashing amongst those households. It is encouraging to know that those who washed their hands with soap between 4 to 6 times also improved during the pandemic compared to before. This may be explained by the heightened WASH campaign during this pandemic era by the health sector and other WASH stakeholders in the country. A study in Indonesia also reported that respondents improved from handwashing of less than 4 times to between 4 to 8 times during the pandemic [18].

Again, the COVID-19 pandemic had some impact on handwashing after sneezing during the pandemic as the percentage of respondents who washed their hands after sneezing during the COVID-19 pandemic saw an increase. This impact of the COVID-19 pandemic on handwashing after sneezing during the pandemic was statistically significant which makes the change attributable to COVID-19. The odds of handwashing after shaking hands with other people were lower during the pandemic than before. This result could be linked to the campaign against handshaking during the pandemic to avoid contamination. As a result, most community members restrained themselves from handshaking and mostly resorted to waiving in most cases. The pandemic could be said to have influenced community members to restrain themselves from exchanging pleasantries through handshakes and rather adopt a new way to relate to friends and family members.

The change that occurred in handwashing after burial suggests that, the pandemic did not improve handwashing behaviours after burial during the pandemic. The above results could be attributed to the fact that at the onset of the COVID-19 pandemic, restrictions were put on burials and social gatherings, until such a time when special arrangements were made for families to organise private burials [16]. As a result, people were prevented from attending burials, especially for people who died of COVID-19 who were buried strictly by the state without the involvement of community members to limit disease transmission.

It is important to highlight that social and behavioral change communication (SBCC) in Ghana at the time of the pandemic is likely to impact handwashing behavior. This is because the research has shown that handwashing improved during the pandemic. In Ghana, during the COVID-19 pandemic, the SBCC included hand hygiene as one of its topics of discussion. A few of the SBCC interventions that enhanced behavioral change with regard to hand hygiene during the COVID-19 pandemic included washing one's hands after visiting the cemetery, after shaking hands with another individual, and after using the restroom. This was evident in the findings of the study, as the handwashing practice during the COVID-19 pandemic improved.

In the fight against the COVID-19 pandemic, investing in WASH is a prudent economic policy for developing countries [2]. The healthcare system must continue to encourage handwashing with the same zeal and commitment once the pandemic is over, not just in the healthcare setting, but also within the communities [16]. In addition to regular sanitation and hygiene education, having community outreach programs that focus on hand hygiene and its risk with certain health outcomes could be a successful strategy for encouraging community members to practice hand hygiene especially washing of hands [18].

**Strengths and limitations:** the study makes a valuable contribution to informing stakeholders and policymakers on the status of use of one of the most effective and recommended interventions against COVID-19 spread such as handwashing among predominantly rural inhabitants in Ghana. It also provides an opportunity for further research on determinants of potential rural-urban handwashing disparity and also willingness and ability to pay for a handwashing facility provided by the government or an investor.

All the measures of this study were based on the responses from individual respondents, which could lead to recall bias and socially desirable responses as some of the respondents may assume, they may be perceived to be doing the wrong things if they do not provide positive responses. We could not independently observe and verify handwashing practices at the community level. Their responses notwithstanding provided further understanding of handwashing behaviours at the community level. In addition to the limitations stated, this study falls short of qualitative analysis. This would have enhanced the findings of the study by complementing the results of the quantitative arm of the study. Another limitation to the study is that the study did not include information on health education and sensitization coverage on the COVID-19 safety etiquette even though this was a major component during the pandemic.

## Conclusion

Handwashing behaviours during the pandemic improved among residents than before. This may be considered as a positive influence on hand hygiene behaviours among community members. Even though the prevalence of ownership of handwashing facilities improved during the pandemic, it is relatively low and needs further improvement. Handwashing with soap and water is not a universal practice among community members and this requires intervention from policy makers. Training on proper handwashing remains very low among rural inhabitants. However, there is a need to intensify health education and media engagement on proper handwashing practices to protect the population against infectious diseases.

### What is known about this topic

- *Handwashing is an effective public health intervention for preventing the spread of infectious diseases such as COVID-19;*
- *Handwashing is among the cheapest and essential interventions against infectious diseases such as diarrhoea, cholera, typhoid, and dysentery*.

### What this study adds

- *Community member´s handwashing practices improved during the pandemic than before;*
- *Television was found to be an appropriate source where the majority received training on handwashing;*
- *Household ownership of handwashing facilities even during the COVID-19 pandemic was low among community members*.
